# Quantitative profiling and diagnostic potential of one-carbon and central metabolism pools in MODY2 and T1DM

**DOI:** 10.1186/s13098-023-01175-x

**Published:** 2023-10-24

**Authors:** Jieying Liu, Ziyan Xie, Junling Fu, Miao Yu, Tong Wang, Cuijuan Qi, Peng Liu, Xiangyi Hui, Dongmei Wang, Lu Ding, Qian Zhang, Ting Xie, Xinhua Xiao

**Affiliations:** 1 China Key Laboratory of Endocrinology of National Health Commission, Diabetes Research Center of Chinese Academy of Medical Sciences, Department of Endocrinology, Peking Union Medical College Hospital, Peking Union Medical College, Chinese Academy of Medical Sciences, No. 1 Shuaifuyuan, Wangfujing Street, Dongcheng District, Beijing, 100730 P. R. China; 2 Department of Medical Research Center, Peking Union Medical College Hospital, Chinese Academy of Medical Sciences & Peking Union Medical College, Beijing, 100730 China; 3 Department of Endocrinology, Beijing Institute of Geriatrics, Xuanwu Hospital, Capital Medical University, Beijing, 100053 China; 4 Department of Endocrinology, The 305 Hospital of People’s Liberation Army of China, Beijing, 100017 China; 5 Department of Endocrinology, Hebei General Hospital, Hebei, 050051 China

**Keywords:** Maturity onset diabetes of the young type 2 (MODY2), Type 1 diabetes, Tricarboxylic acid cycle metabolites, One-carbon metabolism, Diagnostic biomarkers

## Abstract

**Background:**

Maturity-onset diabetes of the young type 2 (MODY2) is a rare genetic disorder characterized as mild fasting hyperglycemia with low risk of vascular complications caused by glucokinase gene mutation. This study aims to investigate metabolites alteration associated with MODY2, exploring possible mechanism underlying characteristic clinical manifestations and low cardiovascular risks of MODY2 and providing serum metabolite biomarkers to facilitating MODY2 diagnosis.

**Methods:**

Fasting serum samples from MODY2, type 1 diabetes (T1DM) and healthy individuals were collected. By using targeted metabolomics via liquid chromatography–tandem mass spectrometry platform, we quantified the metabolites involved in tricarboxylic acid (TCA) cycle and one-carbon metabolism.

**Results:**

Metabolomic profiling revealed significant difference of intermediates from central metabolism cycle, methionine cycle and several amino acids between MODY2 and T1DM groups. Among these, serum citrate, α-ketoglutaric acid, serine, glycine, glutamine and homocysteine were significantly elevated in MODY2 patients compared with T1DM patients; and compared with healthy subjects, malate and methionine levels were significantly increased in the two groups of diabetic patients. The correlation analysis with clinical indexes showed that α- ketoglutarate, serine, glycine, and glutamine were negatively correlated with blood glucose indicators including fasting blood glucose, HbA1c, and GA, while citrate was positively correlated with C-peptide. And homocysteine displayed positive correlation with HDL and negative with C-reactive protein, which shed light on the mechanism of mild symptoms and low risk of cardiovascular complications in MODY2 patients. A panel of 4 metabolites differentiated MODY2 from T1DM with AUC of 0.924, and a combination of clinical indices and metabolite also gained good diagnostic value with AUC 0.948.

**Conclusion:**

In this research, we characterized the metabolite profiles of TCA cycle and one-carbon metabolism in MODY2 and T1DM and identified promising diagnostic biomarkers for MODY2. This study may provide novel insights into the pathogenesis and clinical manifestations of MODY2.

**Supplementary Information:**

The online version contains supplementary material available at 10.1186/s13098-023-01175-x.

## Introduction

Maturity-onset diabetes of the young (MODY) is a kind of rare genetic disorder, constituting 1–5% of all diabetes cases [1, 2]. To date, fourteen subtypes of MODY have been identified, due to mutations of genes that encode transcription factors (e.g., HNF4A and HNF1A), enzymes (e.g., GCK and CEL), and other important molecules (e.g., INS and SUR1), involved in pancreatic cells function and metabolism [3, 4]. Manifestations vary from the causal genes. Among them, glucokinase (GCK)-MODY are the most frequent genotypes, presenting in approximately 10–60% of the cases [5].

GCK-MODY, also named MODY2, exhibits special characteristics, including mild, asymptomatic hyperglycemia accompanied with unique lipid profile, which possess lower level of triacylglycerols (TAGs) and elevated high-density lipoproteins (HDLs) [6–8]. Besides, MODY2 patients possess good clinical prognosis and low risk of diabetic complications compared with T1DM and type 2 diabetes (T2DM) patients [9]. Due to its unique characteristics, MODY2, in comparison to other diabetes, serves as a natural model of cardiovascular protection under concurrent hyperglycemia [7]. Besides, it has been reported that over 80% of patients with MODY are undiagnosed or misdiagnosed as other types of diabetes due to the cross clinical manifestations [10]. Both MODY2 and T1DM patients are early onset without obesity [10], and islet autoantibodies are not sufficient to distinguish MODY and T1DM [11]. Therefore, the phenotype alone and current clinical index are not sufficient for easy differential diagnosis. Cost-effective novel biomarker screening that precedes genetic testing is urgently expected. As both MODY2 and T1DM have young onset ages without insulin resistance, obesity, hypertension and dyslipidemia, and T1DM patients have higher incidence of vascular complications, it will be of great significance for the treatment and prognosis evaluation of diabetes that we can identify these patients early in children and adolescents and explore the underlying mechanism of their characteristic clinical manifestations. The present study chose T1DM as the disease control to investigate the metabolites changes compared to MODY2, revealing the cardiovascular-protective mechanism in MODY2 and identifying early biomarkers may provide new therapeutic targets for diabetes complications.

One-carbon (1-C) metabolites have long been recognized as critical nutrients for growth and development [12]. One-carbon metabolism including folate cycle, methionine cycle, choline metabolism, and transsulfuration pathways, provides one-carbon unit for the biosynthesis and metabolism of nucleic acids, proteins, and lipids, and acts as donor or substrate in epigenetic modification and gene expression [12, 13]. Recently, accumulating evidence has been shown to support the association of one-carbon metabolites with diabetes and cardiovascular diseases (CVD) [14]. The increased plasma homocysteine concentration has been proven to be an independent risk factor for CVD [15, 16]. Amino acids like glycine [17], serine [18], and glutamine [19] have favorable effects in enhancing insulin sensitivity and improving blood glucose homeostasis. High choline concentrations are associated with unfavorable cardiometabolic risk factors (such as: low HDL, high homocysteine, and high BMI) [20], and have a positive correlation with macrovascular disease and cerebrovascular disorders [21].

The tricarboxylic acid cycle (TCA cycle) is a central metabolite pool of amino acid, glucose and fatty acid metabolism, and is crucial to mitochondrial and energy metabolism [22]. Alterations in the TCA cycle have been correlated with numerous pathologies including cardiovascular dysfunction [23] and metabolic syndromes [24]. Intermediates involved in TCA cycle are widely suggested as biomarkers for cardiovascular diseases and diabetes. Lower plasma level of α-Ketoglutarate was found in diabetes and obesity patients [25], and were associated with a higher risk of cardiovascular events [23]. Moreover, circulating levels of succinate, malate and citrate were found related to the risk of cardiovascular disorders (heart failure, hypertension and ischemic heart disease) [26, 27] and metabolic syndromes including type 2 diabetes and obesity [28], indicating the potential role of the TCA cycle in the pathogenesis of cardiovascular outcomes and glucose and lipid metabolism disorders.

Therefore, targeted metabolomics methodology for one-carbon and TCA metabolism has been developed in many epidemiological studies for disease biomarker discovery [29, 30]. So far, very little of noted studies have been conducted on the field of one-carbon and TCA metabolism in MODY2. The aims of this study include: compare differences in one-carbon and TCA metabolites between MODY2, T1DM and healthy controls; investigate the metabolism mechanism of low cardiovascular risks in MODY2 compared with T1DM; explore the diagnostic potential of a serum metabolite biomarkers that could facilitate genetic screening for MODY2. In the present study, we quantified the serum metabolite profiles and identified important metabolites involved in one-carbon and central metabolism pools in MODY2 and T1DM patients, revealing the possible mechanism of favorable prognosis in MODY2 and providing promising biomarkers for distinguishing MODY2 from T1DM.

## Materials and methods

### Study population

The study cohort comprises 97 subjects, including 33 MODY2, 34 T1DM and 30 healthy controls. MODY2 and T1DM patients were recruited from the outpatient clinic of the endocrinology department at the Peking Union Medical College Hospital (PUMCH), Beijing, China, between January 2017 and December 2018. T1DM patients were diagnosed according to the guide of the American Diabetes Association. The inclusion criteria of MODY2 were as follows: the onset age of diabetes of < 45 years; family history of diabetes in at least 2 generations; negative pancreatic islet autoantibodies; nonobese, BMI < 28 kg/m^2^; the *GCK* mutations were verified by Sanger sequencing and genetic analysis (Supplementary Table 1). This study was approved by the ethical standards of the Peking Union Medical College Hospital Ethics Committee and written consent was obtained from all participants.

### Demographic and clinical data collection

Demographic information including age, gender, height, weight, blood pressure, diagnostic age, and family history of diabetes was collected for subsequent analysis. BMI was calculated as weight (kg)/(height (m) ^2^). Venous blood samples of the participants were collected in the morning after fasting for 8–12 h. Fasting plasma glucose (FBG), low-density lipoprotein cholesterol (LDL-c), high-density lipoprotein cholesterol (HDL-C), total cholesterol (TC), triglyceride (TG), and hsCRP were measured by an automatic biochemical analyzer. Fasting C-peptide were assayed by chemiluminescent analysis. Glycated albumin (GA) and glycated hemoglobin A1c (HbA1c) was analyzed by high-performance liquid chromatography.

### Sample preparation

The quantification process was as previously reported [31, 32]. Firstly, standard solutions were dissolved in water and stored at − 20 °C. The stock solutions were diluted serially with 2% ACN (acetonitrile: water, v:v = 2:98) to generate working solutions. Standard solutions were prepared with concentration range of 1- 1000ng of L-threonine (Thr), L-glutamic acid (Glu), L-cysteine (Cys), L-Glutamine (Gln), L-serine (Ser), L-methionine (Met), S-Adenosylhomocysteine (SAH), S-adenosylmethionine (SAM), glycine, Betaine, Trimethylamine, homocysteine, Cystathionine, Glutathione, Fumaric acid, Succinic acid, α-KG, Pyruvate, Citrate, Lactic, Malate and Oxaloacetate. Secondly, [13 C, 15 N]-labeled amino acid mixture (Sigma-Aldrich, USA) was diluted 100 times with 2% ACN (acetonitrile: water, v:v:v = 98:2) as IS working solution. QC sample was acquired for every 10 samples to further monitor the stability of the methods. LC-MS/MS analysis. Plasma sample (50 µL) was mixed with 10 µL isotope-labeled standard. 150 µL pre-cooled organic solvent (acetonitrile: methanol, v:v = 50:50) was added to the mixture and vortex mixed evenly. Mixed liquid was centrifuged at 14,000 g for 15 min at 4 °C. The supernatant is dried in the centrifuge concentrator (Labconco Centrivap) and re-suspended with 50 µL 2% ACN (acetonitrile: water, v:v = 2:98) and transferred to sample vials for LC-MS/MS analysis.

### Metabolomic analysis

LC and MS conditions LC-MS/MS analysis was conducted on ExionLC AD consisting of binary pumps, an on-line degassing unit, an autosampler, and a column oven (Shimadzu Corporation, Kyoto, Japan), which is coupled with an AB Sciex 6500 + QTRAP mass spectrometer consisting of an electrospray ionization (ESI) source (AB SCIEX, Framingham, MA, USA). Chromatographic separation was achieved on Waters Acquity UPLC HSS T3 Column, 100Å, 1.8 μm, 2.1 mm X 100 mm maintained at 40 °C, at a flow rate of 0.3 mL/min. The mobile phases consisted of Solution A (0.1% Formic acid in water) and Solution B (100% acetonitrile). The following gradient was used: 0–1 min, 98% A; 1–5 min, 98%-45% A; 5–8 min, 45%-0% A; 8–13 min, 100% A; 13-13.1 min, 100%-2% A; 13.1–18 min, 98% A, with a total run time of 18 min. The ion source was operated in mix mode: curtain gas, 35 psi; nebulizer gas 50 psi; auxiliary gas 50 psi; ion spray voltage, 5500 V/-4500 V (positive/negative); and temperature 500 °C. Multiple reaction monitoring (MRM) transitions were identified for all analyses and isotope-labeled standard. Data acquisition and analysis were all performed with Analyst 1.6.3 software (AB SCIEX) and OS software (AB SCIEX).

### Data processing and statistical analysis

We first conducted PCA analysis using prcomp function in R for dimension reduction of metabolomic data. Multivariate analysis was conducted using partial least squares regression discriminant analysis (PLS-DA) in SIMCA version 14.1 (MKS Umetrics AB, Umea, Sweden). Then the normalized concentration of metabolites in each sample were presented in the heatmap using the R package ComplexHeatmap. Statistical analyses were performed using Statistical Package for the Social Sciences (SPSS 26.0, Chicago, IL, USA). Normally distributed continuous variables were described as mean ± SD and compared by the paired t-test (2 groups) and ANOVA (Bonferroni’s post hoc test). Categorical variables were expressed as percentage and analyzed by the chi-square test. The principal component analysis (PCA) model was used to analyze the overall distribution of each sample. Correlation between clinical indices and serum metabolites were analyzed by the partial Spearman’s correlation test adjusted for age and sex, and only associations with p < 0.05 were indicated. Receiver operating characteristic (ROC) curves were conducted to identify the optimal cutoff values for metabolic performance between MODY2 and T1DM. The ROC analysis of combination of multiple metabolites is based on a logistic regression model. The area under the curve (AUC) was used as a measure of overall performance.

## Results

### Baseline characteristics of study populations

In the present study, there were 97 participants, comprising 33 patients with MODY2, 34 patients with T1DM and 30 healthy controls. The characteristics of all the subjects are summarized in Table 1. No significant difference in gender, age, BMI and blood pressure was observed between controls, MODY2 and T1DM. The blood glucose profiles including fasting glucose (p < 0.0001), GA (p < 0.0001) and HbA1c (p < 0.0001) were found increasing progressively from control to MODY2 and T1DM. The level of fasting C-peptide was significantly decreased (p < 0.0001) whereas the fasting insulin was elevated in T1DM patients compared with both MODY2 and control (p = 0.0034), indicating the islets dysfunction and insulin treatment in T1DM patients. The lipid profiles displayed cardioprotective effects in MODY2 group. In MODY2 patients, the LDL-c level was lower than T1DM and comparable to the healthy control (p = 0.016), while HDL-c in MODY2 group was significantly increased (p = 0.0002). There was no significant difference in TG and TC among the three groups. High sensitivity C-reactive protein (hsCRP) was significantly elevated in T1DM relative to both MODY2 and controls (p = 0.0179).

**Table 1:** Demographic and clinical characteristics of the study subjects

Parameters	MODY2 (n = 33)	T1DM (n = 34)	Control (n = 30)	*P*
Gender (M/F)	12/21	14/20	14/16	0.7088
Age (Year)	24.5 ± 15.83	19.1 ± 9.94	19.6 ± 11.18	0.2099
Birth weight (kg)	3.1 ± 0.45	3.5 ± 0.60	3.4 ± 0.49	0.074
Height (cm)	151.5 ± 21.13	160.1 ± 15.03	153.6 ± 11.98	0.094
Weight (kg)	46.4 ± 19.82	49.9 ± 17.38	46.9 ± 12.35	0.669
BMI (kg/m2)	19.1 ± 4.44	19.0 ± 4.97	19.6 ± 3.11	0.8635
Systolic blood pressure (mmHg)	105.3 ± 12.93	113 ± 15.95	108 ± 6.36	0.142
Diastolic blood pressure (mmHg)	66.2 ± 10.29	72.5 ± 11.05	71 ± 7.21	0.086
Fasting glucose (mmol/L)	6.8 ± 0.57	8.8 ± 3.48 *	4.8 ± 0.37 **#**	**< 0.0001**
Fasting insulin (mIU/L)	6.5 ± 3.48	26.5 ± 42.00 *	8.0 ± 3.82 **#**	**0.0034**
Fasting C-peptide(ng/ml)	1.03 ± 0.45	0.40 ± 0.32 *	1.12 ± 0.42	**< 0.0001**
HbA1c (%)	6.3 ± 0.37	8.7 ± 2.29 *	4.7 ± 0.4 **#**	**< 0.0001**
GA (%)	18.0 ± 1.46	23.5 ± 6.39 *	13.6 ± 1.19 **#**	**< 0.0001**
Triglyceride (mmol/L)	0.6 ± 0.30	0.7 ± 0.43	0.8 ± 0.50	0.080
Total cholesterol (mmol/L)	4.4 ± 0.77	4.5 ± 0.86	4.0 ± 0.67	0.087
LDL-C (mmol/L)	2.1 ± 0.57	2.6 ± 0.76 *	2.2 ± 0.68	**0.016**
HDL-C (mmol/L)	1.6 ± 0.26	1.3 ± 0.23 *	1.5 ± 0.21	**0.0002**
Creatinine (umol/L)	55.9 ± 18.10	52.6 ± 16.64	53.6 ± 11.32	0.704
Uric acid (umol/L)	258.7 ± 73.32	255.6 ± 64.20	307.5 ± 93.07	**0.032**
High-sensitivity C-reactive protein (mg/L)	0.3 ± 0.27	1.46 ± 2.38 *	0.7 ± 1.30	0.0179

The data were expressed as mean ± standard deviation after one-way ANOVA;

Non-normally distributed data were expressed as the median (25th-75th)

P < 0.05 indicates a significant difference, which is indicated in bold. Differences between MODY2 and T1DM are indicated by *, and differences between MODY2 and control are indicated by #

### Metabolomics profile in the study participants

Enrolled participants included 33 diagnosed MODY2 patients, 34 T1DM patients and 30 age- and sex-matched healthy controls who underwent metabolomic profiling. Serum was collected from all the participants and used the target LC-MS/MS approach to analyze the metabolomic profiles. Briefly, the optimized MRM transitions and parameters of the standards were developed. The MRM parameters of standards including the transitions, decluttering potential (DP), Collision energy (CE), and linearity. All the standards exhibited excellent linearity with R^2^ greater than 0.99 (Supplementary Table 2).

**Fig. 1:**
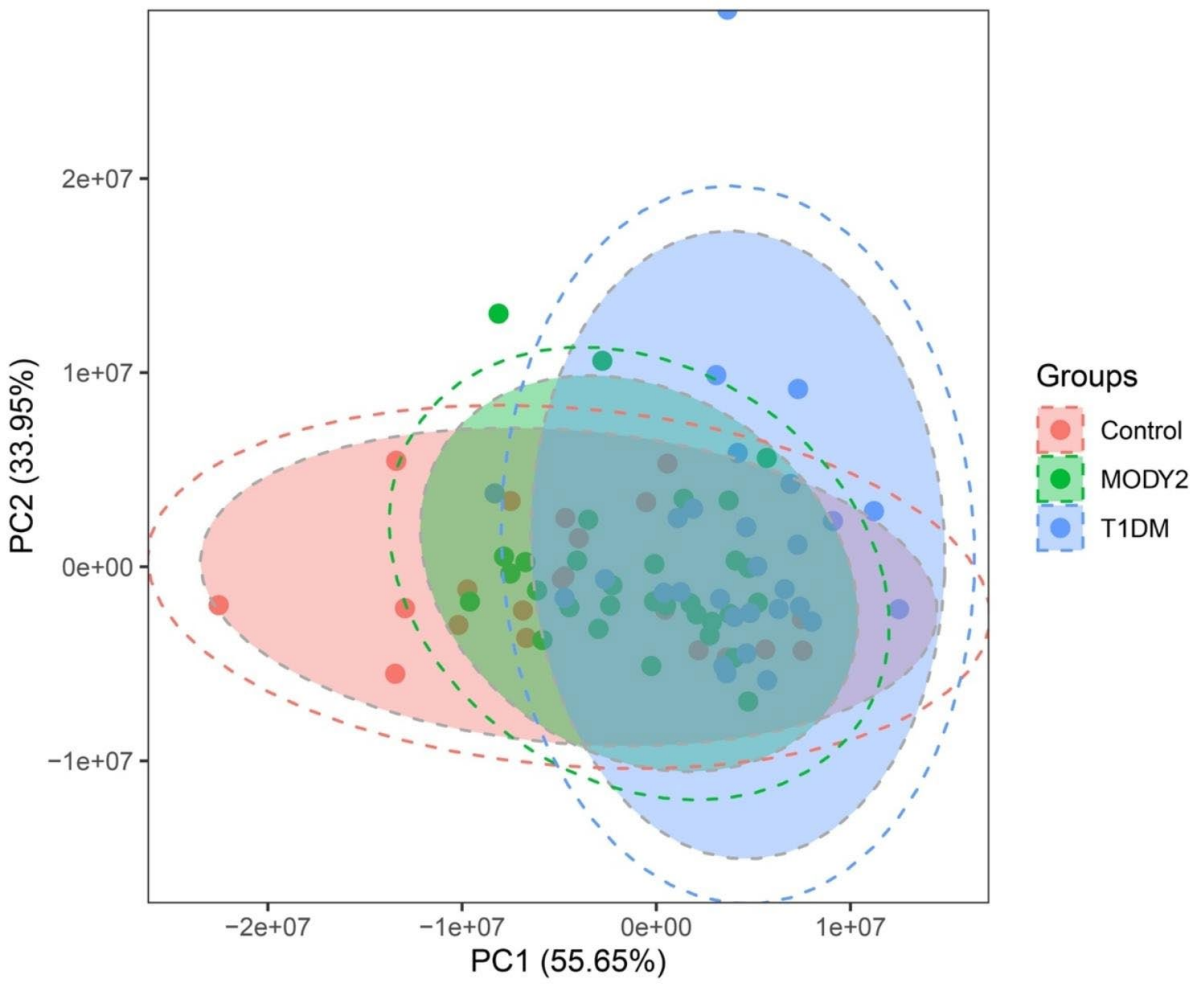
Principle component analysis (PCA). PCA plots show separation of metabolite profiles among MODY2, T1DM and control groups. Areas of 95% confidence are highlighted in red, green, and blue, respectively

**Fig. 2:**
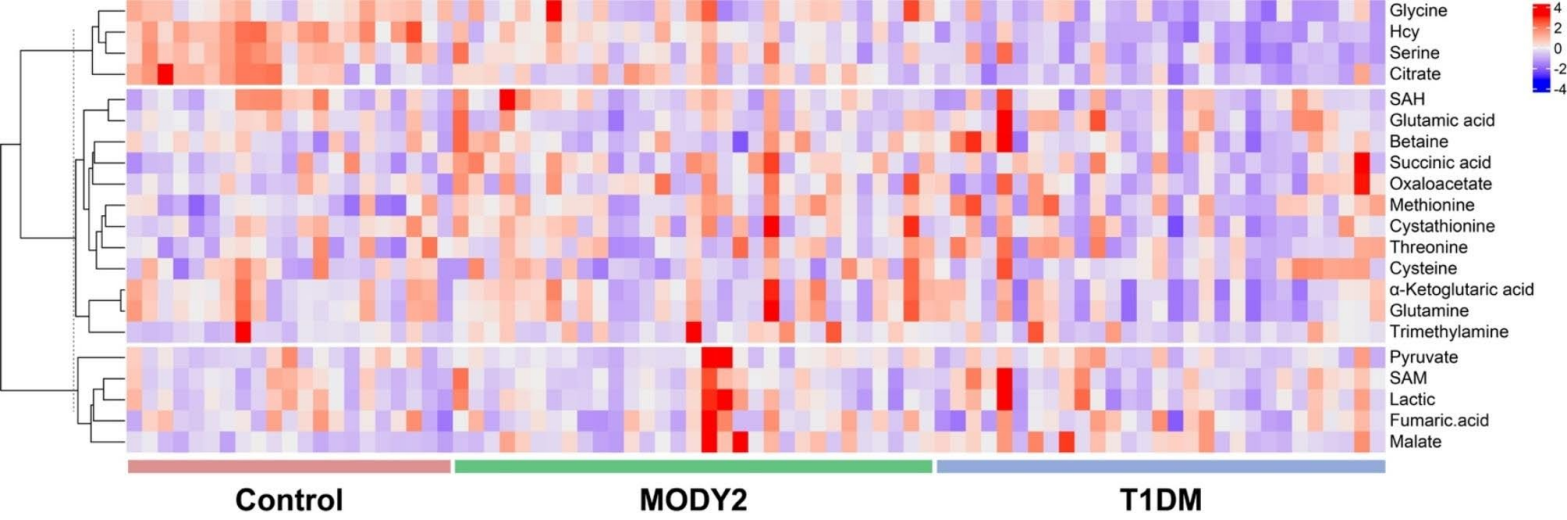
Heatmap showing the hierarchical clustering characteristics of metabolites. The color intensity indicates the corresponding abundance difference. Blue represents decreasing expression, and red indicates increasing expression

PCA analysis was utilized to characterize the differential profiling between MODY2, T1DM and control. As shown in Fig. 1, the T1DM group was distributed separately from the control, and T1DM and MODY2 groups were separated to some extent, while the MODY2 cluster was tended to be overlapped with the control group, which indicated that the serum metabolites of MODY2 were almost comparable to nondiabetic healthy individuals. PLS-DA analysis (Supplementary Fig. 1) also obtained good discrimination among three groups. Heatmap showed the hierarchical cluster analysis of the screened metabolites (Fig. 2). A total of 21 metabolites were identified, including one-carbon metabolism-related amino acids (serine, glycine, glutamine, etc.), intermediates in TCA cycle (pyruvate, α-Ketoglutaric acid, citrate, etc.), metabolites involved in methionine cycle (methionine, homocysteine, S-Adenosyl methionine and S-adenosylhomocysteine) and trans-sulfuration pathway (cysteine and cystathionine). The hierarchical cluster analysis revealed 3 distinct patterns of metabolites among groups (Fig. 2). Some metabolites were decreasing progressively from control to MODY2 and T1DM, such as homocysteine and citrate. A cluster of metabolites increased in MODY2 and control while reduced in T1DM, such as α-ketoglutaric acid and glutamine. Some metabolites were enriched in MODY2 compared with control and T1DM, for example, pyruvate and malate. Among these, glycine, serine, glutamine, citrate, α-ketoglutaric acid, succinate, malate, methionine, and homocysteine displayed significant differences between groups.

### One-carbon and TCA cycle intermediates alteration

**Fig. 3:**
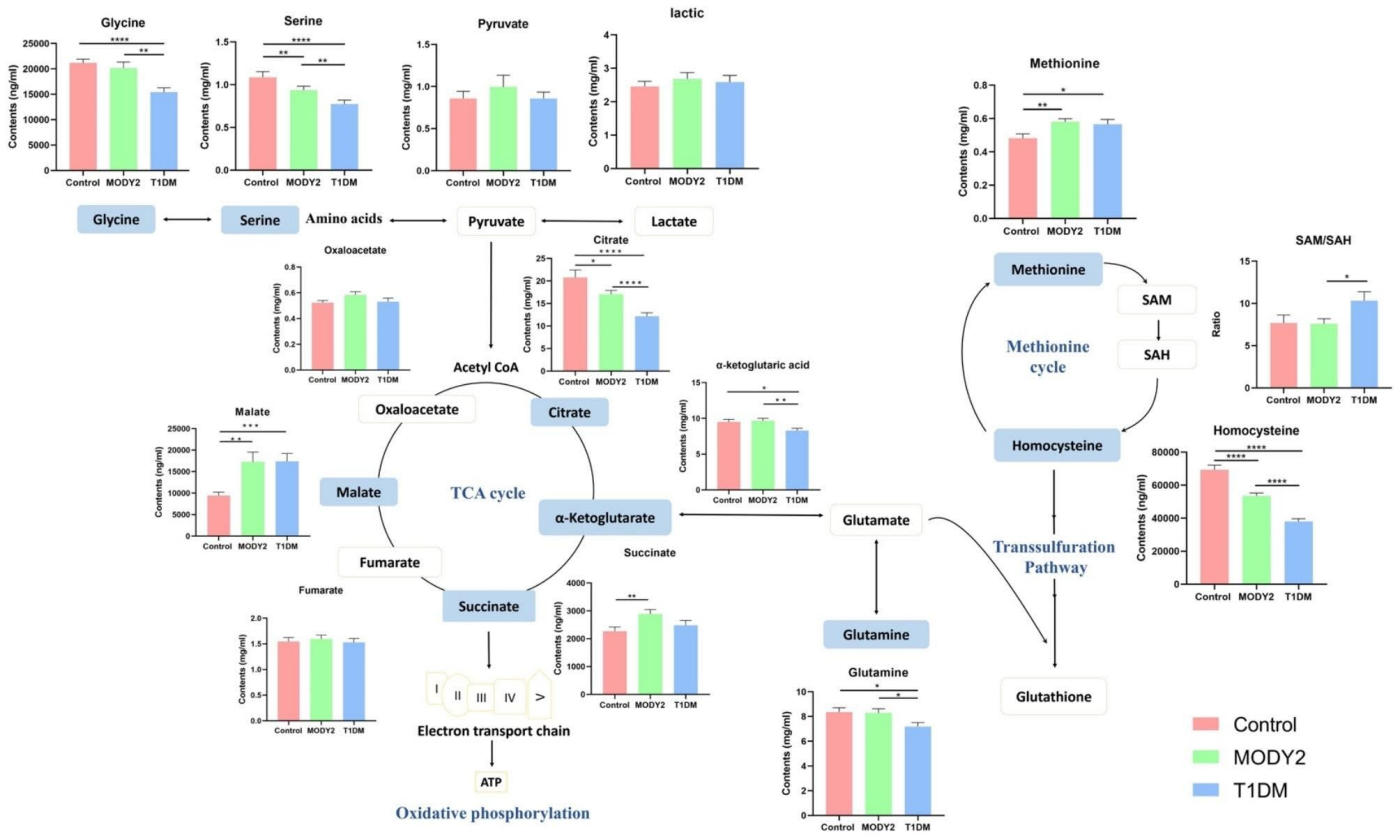
Alteration of TCA cycle and one-carbon metabolism among three groups. Data are represented as mean ± SD. ****P ≤ 0.0001, ***P ≤ 0.001, **P ≤ 0.01, *P ≤ 0.05 between groups via Bonferroni’s test

As shown in Fig. 3, serum TCA intermediates including citrate, α-Ketoglutarate, succinate and malate were significantly differentiated between groups. Citrate (p < 0.0001) and α-Ketoglutarate (p = 0.0116) were decreased in T1DM group compared to control and MODY2. The level of succinate was found significantly increased in MODY2 (p = 0.0309) whereas malate was enriched in both MODY2 and T1DM relative to control (p = 0.0109). The levels of pyruvate and lactate were not different between groups. Besides, TCA cycle related amino acids were also fluctuated. T1DM had lower serum concentrations of serine (p < 0.0001), glycine (p = 0.0002) and glutamine (p = 0.0216) compared with MODY2 and control. In addition, intermediates involved in methionine cycle were differentially expressed between groups. The level of methionine tended to be higher in MODY2 and T1DM groups compared with healthy control (p = 0.0214), while homocysteine tended to be lower in diabetes groups relative to control (p < 0.0001). And homocysteine was significantly decreased in T1DM group compared to MODY2 (p < 0.0001). S-Adenosyl methionine (SAM) and S-adenosylhomocysteine (SAH) showed no difference among groups, but the ratio of SAM to SAH was significantly decreased in MODY2 patients compared with T1DM (p = 0.0204). Besides, to exclude the potential impact of poor blood glucose control on metabolites, we further conducted stratified analyses according to fasting blood glucose and HbA1C, and T1DM patients were classified as T1DM^a^ (blood glucose similar with MODY2 patients, well-controlled group) and T1DM^b^ (poor blood glucose control group). Similar results were seen in the metabolites alterations among MODY2, T1DM and control groups before and after stratified analyses, which successfully eliminated the potential effects of blood glucose on metabolites (Supplementary Fig. 2).

### Correlations between metabolites and clinical characteristics

**Fig. 4:**
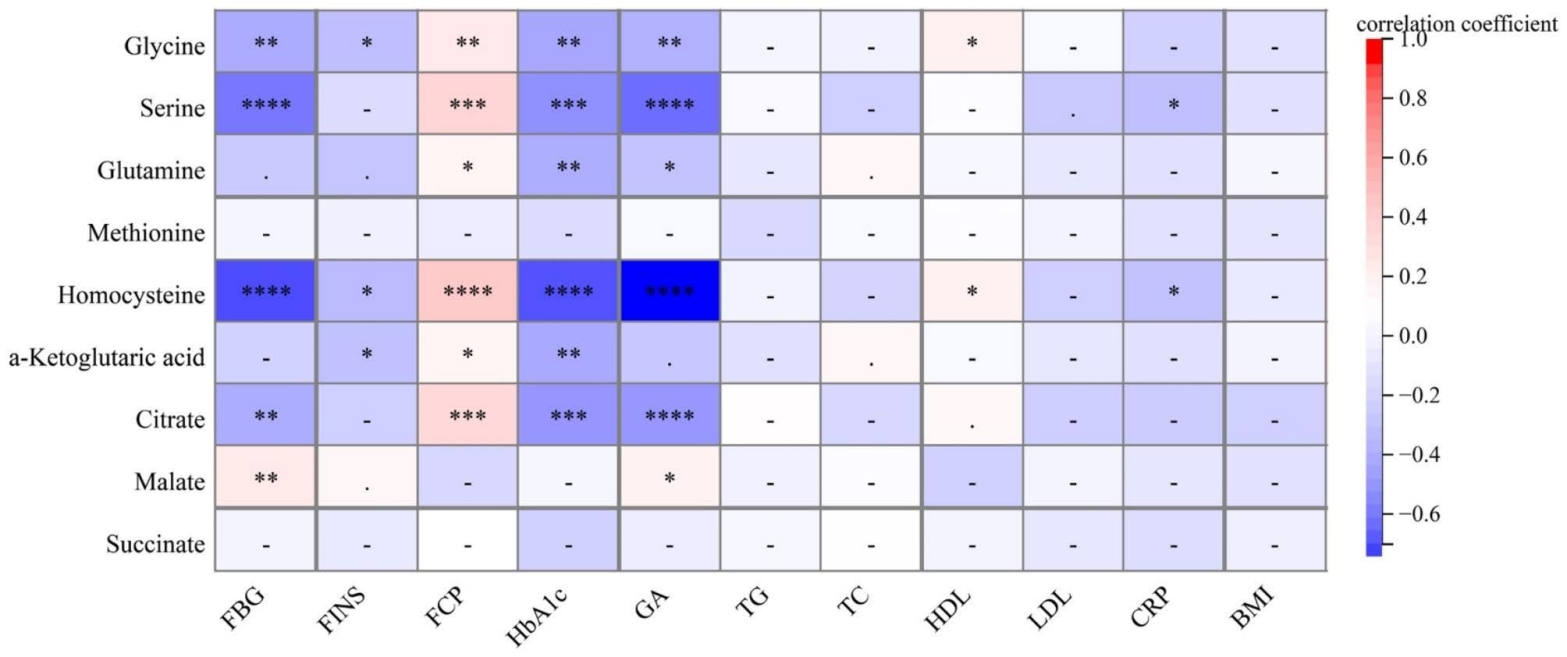
Correlation matrices of serum metabolites and clinical indices. The correlations between serum metabolites and clinical indices were analyzed by Partial Spearman correlation analysis was adjusted for age and sex. Color indicates correlation coefficient (red representing positive correlations; blue representing negative correlations), and color intensity presents strengths of correlation (darker color indicates stronger correlation). Spearman’s correlation coefficient was shown if p < 0.05

The correlations between differential metabolites among three groups and clinical indicators were further analyzed (Fig. 4). We observed strong negative associations between glycine [r(FBG)= -0.35, p < 0.01; r(HbA1c)=-0.37, p < 0.05; r(GA)=-0.32, p < 0.01], serine [r(FBG)= -0.54, p < 0.0001; r(HbA1c)=-0.46, p < 0.001; r(GA)=-0.57, p < 0.0001], glutamine [r(HbA1c)=-0.34, p < 0.01; r(GA)=-0.23, p < 0.05], homocysteine [r(FBG)=-0.64, p < 0.0001; r(HbA1c)=-0.63, p < 0.0001; r(GA)=-0.70, p < 0.0001], α-Ketoglutarate [r(HbA1c)=-0.36, p < 0.01], citrate [r(FBG)=-0.34, p < 0.01; r(HbA1c)=-0.44, p < 0.001; r(GA)=-0.43, p < 0.0001] and glucose profiles including fasting glucose, GA and HbA1c, suggesting that these metabolites may elicit favorable effects against hyperglycemia. On the contrary, malate showed positive relation with fasting glucose (r = 0.30, p < 0.01) and GA (r = 0.24, p < 0.05). To this extent, glycine (r = 0.30, p < 0.01), serine (r = 0.42, p < 0.001), glutamine (r = 0.22, p < 0.05), homocysteine (r = 0.49, p < 0.0001), α-Ketoglutarate (r = 0.22, p < 0.05) and citrate (r = 0.39, p < 0.001) were positively correlated with fasting C-peptide, indicating the islet function-protective roles of these metabolites. The levels of serum insulin maybe influenced by insulin treatment of patients; therefore, fasting C-peptide was more representative of islet function. Besides, glycine (r = 0.25, p < 0.05) and homocysteine (r = 0.25, p < 0.05) showed positive relations with HDL, which implied that increased glycine and homocysteine maybe lipid favorable under hyperglycemia. Furthermore, serine and homocysteine displayed significantly negative correlation with CRP, suggesting that enhanced levels of serine (r=-0.25, p < 0.05) and homocysteine (r=-0.23, p < 0.05) maybe related to low risk of cardiovascular disorders in this population.

### Differentially diagnostic value of metabolite biomarkers

**Fig. 5:**
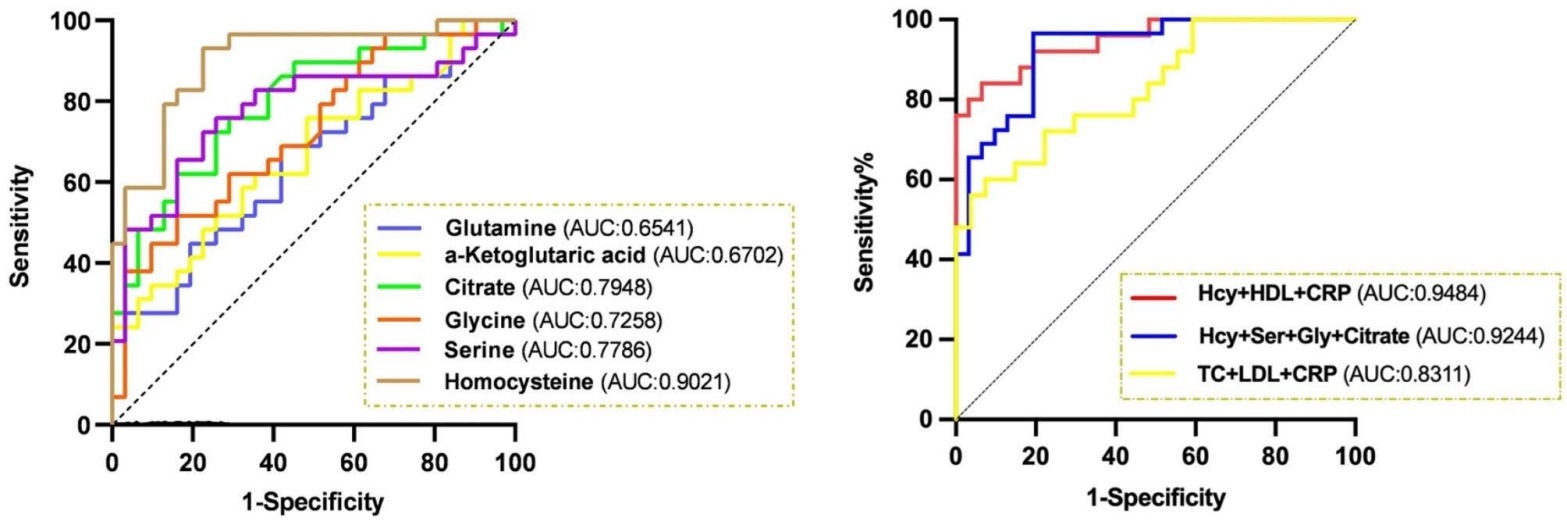
ROC curves of different metabolites for differentiating MODY2 from T1DM.

The individual discriminating performance of these metabolite biomarkers was further supported by classical univariate receiver operating characteristic (ROC) analysis (Fig. 5). The results suggested that homocysteine, glycine, serine and citrate possessed good differential diagnostic value for T1DM and MODY2. When distinguishing MODY2 from T1DM, the AUC of homocysteine was 0.9021, which permitted 93.1% sensitivity and a specificity of 77.42%, indicating high diagnostic capability as biomarkers. The AUC of citrate for distinguishing MODY2 from T1DM was 0.7948, which yielded 75.86% sensitivity and 70.97% specificity. The AUC of serine was 0.7786, allowing 72.41% sensitivity and 77.42% specificity whereas glycine led to an AUC of 0.7258 with 62.07% sensitivity and 70.97% specificity.

In addition, we examined the differential value of the combination of metabolites and clinical parameters to discriminate MODY2 from T1DM. In this study, the AUC of the combination of HDL, hsCRP and homocysteine was 0.9484, with a sensitivity of 84.00% and a specificity of 93.55%, which significantly improved the accuracy of distinguishing MODY2 and T1DM compared to homocysteine alone. Besides, we also analyzed the discriminating performance of the combination of homocysteine, citrate, serine and glycine. The combination of these metabolites led to an AUC of 0.9244, exhibiting 96.55% sensitivity and 80.65% specificity. Our previous work [33] found that a model consist of CRP, TC and LDL could be used to predict MODY subtypes more effectively than the individual indexes. Therefore, we also examined the diagnostic value of the combination CRP, TC and LDL in this study, and the AUC was 0.8311 yielding 76% sensitivity and 66.7% specificity. Taken together, our results showed that a panel of four serum metabolites and combination of metabolite and clinical indexes exhibited satisfactory performance compared with existing model in differentiating MODY2 from T1DM patients, indicating the potential of serum metabolic markers as a preceding test to facilitate the correct diagnosis of MODY2.

## Discussion

Although MODY2 is a common type of monogenic diabetes and *GCK*-point mutation remains the major cause, there is a lack of knowledge about the favorable clinical characteristics and low cardiovascular risk of MODY2. Besides, due to the high rates of misdiagnosis, identification of efficient and practical biomarkers measured by high-throughput methods for differentiating diagnosis and pathogenesis mechanism of MODY2 are of great significance. In this study, we found that the serum levels of metabolites involved in one-carbon and central metabolism pools of MODY2 were unique compared to T1DM and control. Among these, we identified a potential diagnostic panel for distinguishing MODY2 from T1DM using a combination of 4 metabolites including homocysteine, citrate, serine and glycine according to the ROC analysis. Furthermore, this study also found that combination of clinical indices (HDL and hsCRP) and metabolites (homocysteine) allowed a better discrimination between MODY2 and T1DM.

In the present study, we showed that TCA cycle intermediates were significantly altered among MODY2, T1DM and healthy control. Growing evidence has indicated that dysregulations of the TCA cycle and energy flux were associated with multiple pathological status related to oxidative stress including inflammation, insulin resistance and cardiovascular disorders [22], and several intermediates of TCA cycle have been considered as cardiovascular biomarkers. In our study, serum citrate was significantly decreased, and malate was increased in two diabetic groups compared to healthy control, and we also found that citrate and α-ketoglutarate were significantly reduced in T1DM compared with MODY2. Studies have shown that low plasma citrate and α-ketoglutarate were correlated with high risk of cardiovascular outcomes such as coronary artery disease, myocardial infarction and atrial fibrillation [34], while higher level of malate was associated with increased incidence of atrial fibrillation [23]. Both clinical study and animal experiments [25, 35, 36] have showed that α-ketoglutarate exerted favorable effects on glucose metabolism and obesity, and serum α-ketoglutarate was inversely correlated with diabetic biomarker (HbA1c). Our correlation analysis was consistent with previous reports that citrate and α-ketoglutarate were negatively associated with glucose indicators including fasting blood glucose, HbA1c and GA. And we also found that citrate had positive correlation with C peptide indicating that decreased citrate was associated with alterations in β cell function. The metabolites abundance was consistent with the clinical manifestations that compared with MODY2, T1DM individuals have more severe hyperglycemia and worse islet dysfunction; and explained the non-obesity and lower risk of cardiovascular complications in MODY2.

In addition, our results showed that succinate was increased in MODY2 patients compared to control. Elevating level of succinate can be physiological or pathological [37, 38]. Succinate acts an important role in immunity response via binding its receptor succinate receptor 1 (SUCNR1) [39]. A recent study proposed that succinate–SUCNR1 signaling may play dual role as inflammatory or anti-inflammatory mediator depending on the status of cells expressing SUCNR1 [40]. Succinate promotes anti-inflammatory response through activating SUCNR1 in the adipose tissue from healthy individuals, whereas the expression of SUCNR1 was decreased in obese patients and succinate shifted to a pro-inflammatory role [41]. Therefore, whether succinate plays a harmful or protective role in MODY2 remains further exploration and SUCNR1 level need to be measured. However, our findings indicate a potential link between GCK and inflammation via succinate–SUCNR1 pathway.

This study also found that methionine cycle was significantly differed among groups. Methionine cycle catabolizes and regenerates methionine, which serves as an important part in one-carbon metabolism. The methionine cycle generates SAM, which could donate a methyl group to target molecules and becomes SAH, and subsequently homocysteine [42]. Homocysteine then undergoes re-methylation to regenerate methionine [12]. This cycle is crucial for histone methylation and DNA methylation reactions [42]. The 4 C study reported that serum methionine served as a predictive biomarker for T2DM which was positively correlated with the incidence of T2DM in normoglycemic subjects [43]. Shaghayegh et al. [44] also supported that methionine was positively associated with diabetes. In the present study, methionine displayed a significant elevation in diabetic groups compared with healthy controls, which was consistent with the previous studies.

Besides, we also revealed that homocysteine decreased progressively from control to MODY2 and T1DM. And plasma homocysteine possessed good value in discriminating MODY2 from T1DM, with an AUC of 0.9021. Commonly, increased plasma homocysteine is considered as an independent risk factor for coronary artery disease, peripheral vascular disorder and thrombosis [45]. However, reduced circulating homocysteine have been reported in some populations with T1DM (from children to adults) compared with control [46–48] and plasma homocysteine showed a positive correlation with age in diabetic patients [47]. Therefore, in some populations with young age, the low concentration of homocysteine (under the threshold of 15µM) may not statistically represent the risk of cardiovascular events but acts as diagnostic biomarkers for distinguish MODY2 and T1DM, though the possible mechanism remains to be explored. Moreover, the decreased homocysteine and accumulated methionine in our findings probably indicated the enhanced homocysteine re-methylation in diabetic patients (MODY2 and T1DM), which is an important process in epigenetic modification, as methionine is the substrate for synthesizing SAM [12]. The ratio of SAM to SAH represents the methylation capacity and changes in SAM/SAH then impact many methylation reactions [49]. Our results showed that ratio of SAM/SAH was significantly altered in T1DM when compared with both MODY and control groups. This impressed us that T1DM might associated with metabolism remodeling process and might explain the high prevalence of complications among these patients. Moreover, GCK mutation may influence methylation reactions by altering the methionine cycle, and further experiments were required to validate the association between GCK and key enzymes involved in methionine cycle.

Furthermore, amino acids including serine, glycine and glutamine, which are related to TCA cycle and one-carbon metabolism were changed between groups. Accumulating evidence identifies serine and glycine participate in the etiology of multiple metabolic diseases and dietary supplementation of these two amino acids exerts beneficial effects on glucose homeostasis and diabetes-related complications [17, 18, 50]. Low plasma glycine has been consistently reported in obesity, diabetes and NAFLDs [51, 52]. The concentrations of glycine and serine are positively associated with insulin sensitivity and secretion whereas inversely correlated with insulin resistance [18]. Similarly, glutamine has been suggested as a biomarker for obesity and diabetes, which is negatively associated with BMI and HOMA-IR index [53]. And a systemic increase in glutamine levels alleviates inflammation and improves peripheral insulin sensitivity such as skeletal muscle and adipose tissue [53]. In the present study, all three amino acids were significantly reduced in T1DM group, while the concentrations of glycine and glutamine in MODY2 were similar to those in normal controls, thus providing evidence for the better glucose homeostasis, insulin sensitivity and less inflammation of MODY2 patients.

Additionally, the present study identified a panel including 4 metabolites (homocysteine, citrate, serine and glycine) possessed satisfactory performance in distinguishing MODY2 from T1DM; and the combination of metabolite (homocysteine) and clinical indexes (HDL and hsCRP) exhibited better diagnostic value than a single index and existing model, suggesting the potential of serum metabolite biomarkers as a high-throughput method to provide evidence for implementing genetic testing, thus facilitating diagnosis of MODY2 during clinical practice.

Our findings are subject to certain limitations. Firstly, our observations were only based on a Chinese population. Thus, validation in other racial and ethnic populations is required. Secondly, our research was cross-sectional, therefore further prospective study and animal experiments are needed to clarify the casual association between these metabolites and diabetes-related cardiovascular complications. Thirdly, how individual *GCK* mutations affects the metabolites awaits further elucidation. Finally, due to the very low prevalence of MODY2, the sample size may be insufficient to build a confirmatory cohort in this study. Other study working on “omics” of MODY2 could provide external evidence supporting our findings. Isabel et al. [54] compared the bacterial flora in T1DM and MODY2 cohorts of Caucasian origin and found that T1DM gut microbiota profiling was associated with inflammation and autoimmune response, while gut microbiota in MODY2 has a dominant role of succinate-producing, which is consistent with our results that the succinate level was significantly increased in MODY2 and anti-inflammatory metabolites were reduced in T1DM. However, larger scale and multi-racial studies are still required to validate the capability of metabolite biomarkers in clinical practice.

## Conclusion

To summarize, our findings characterized serum metabolites related with one carbon and central metabolism pools in MODY2 and T1DM patients and proposed possible mechanism of mild symptom and low vascular complications in MODY2 patients. To our knowledge, this is the first study assessing TCA and one carbon-related metabolites in MODY2. The identification of a discriminatory panel consisting of 4 metabolites is promising for providing added grounds for genetic testing implementation to facilitate the correct diagnosis of MODY2. Although these findings need further replications in other populations, our study extends the current knowledge on MODY2 diagnosis and pathogenesis.

### Electronic supplementary material

Below is the link to the electronic supplementary material.

Supplementary Material 1

## Data Availability

The datasets generated and analyzed during the current study are available from the corresponding author on request. Individual data cannot be shared for reasons of patient privacy.

## Abbreviations

MODY2: Maturity-onset diabetes of the young type 2
T1DM: Type 1 diabetes
TCA: Tricarboxylic acid
FBG: Fasting plasma glucose
TAGs: Triacylglycerols
HDLs: High-density lipoproteins
LDL-c: Low-density lipoprotein cholesterol
TC: Total cholesterol
GA: Glycated albumin
HbA1c: Glycated hemoglobin A1c
hsCRP: High sensitivity C-reactive protein
ROC: Receiver operating characteristic
AUC: Area under the curve

## References

[1] Kim SH (2015). Maturity-onset diabetes of the Young: what do Clinicians need to know?. Diabetes Metab J.

[2] Misra S, Owen KR (2018). Genetics of Monogenic Diabetes: Present Clinical Challenges. Curr Diab Rep.

[3] Jang KM (2020). Maturity-onset diabetes of the young: update and perspectives on diagnosis and treatment. Yeungnam Univ J Med.

[4] Braverman-Gross C, Benvenisty N (2021). Modeling Maturity Onset Diabetes of the Young in Pluripotent Stem cells: Challenges and Achievements. Front Endocrinol (Lausanne).

[5] Bishay RH, Greenfield JR (2016). A review of maturity onset diabetes of the young (MODY) and challenges in the management of glucokinase-MODY. Med J Aust.

[6] Fajans SS, Bell GI, Polonsky KS (2001). Molecular mechanisms and clinical pathophysiology of maturity-onset diabetes of the young. N Engl J Med.

[7] Wang X, Lam SM, Cao M, Wang T, Wang Z, Yu M (2021). Localized increases in CEPT1 and ATGL elevate plasmalogen phosphatidylcholines in HDLs contributing to atheroprotective lipid profiles in hyperglycemic GCK-MODY. Redox Biol.

[8] Spégel P, Ekholm E, Tuomi T, Groop L, Mulder H, Filipsson K (2013). Metabolite profiling reveals normal metabolic control in carriers of mutations in the glucokinase gene (MODY2). Diabetes.

[9] Steele AM, Shields BM, Wensley KJ, Colclough K, Ellard S, Hattersley AT (2014). Prevalence of vascular complications among patients with glucokinase mutations and prolonged, mild hyperglycemia. JAMA.

[10] Liu L, Liu Y, Ge X, Liu X, Chen C, Wang Y et al. Insights into pathogenesis of five novel GCK mutations identified in chinese MODY patients. Metabolism. 2018;89. 10.1016/j.metabol.2018.09.004 30257192

[11] Urbanová J, Rypáčková B, Procházková Z, Kučera P, Cerná M, Anděl M (2014). Positivity for islet cell autoantibodies in patients with monogenic diabetes is associated with later diabetes onset and higher HbA1c level. Diabet Med.

[12] Ducker GS, Rabinowitz JD (2017). One-Carbon Metabolism in Health and Disease. Cell Metab.

[13] Mentch SJ, Locasale JW (2016). One-carbon metabolism and epigenetics: understanding the specificity. Ann N Y Acad Sci.

[14] Raghubeer S, Matsha TE. Methylenetetrahydrofolate (MTHFR), the One-Carbon cycle, and Cardiovascular Risks. Nutrients. 2021;13(12). 10.3390/nu13124562 PMC8703276 34960114

[15] Virtanen JK, Voutilainen S, Alfthan G, Korhonen MJ, Rissanen TH, Mursu J (2005). Homocysteine as a risk factor for CVD mortality in men with other CVD risk factors: the Kuopio Ischaemic Heart Disease Risk factor (KIHD) study. J Intern Med.

[16] Jamaluddin MS, Yang X, Wang H, Hyperhomocysteinemia (2007). DNA methylation and vascular disease. Clin Chem Lab Med.

[17] Alves A, Bassot A, Bulteau A-L, Pirola L, Morio B. Glycine metabolism and its alterations in obesity and metabolic Diseases. Nutrients. 2019;11(6). 10.3390/nu11061356 PMC6627940 31208147

[18] Holm LJ, Buschard K (2019). L-serine: a neglected amino acid with a potential therapeutic role in diabetes. APMIS.

[19] Darmaun D, Torres-Santiago L, Mauras N (2019). Glutamine and type 1 diabetes mellitus: is there a role in glycemic control?. Curr Opin Clin Nutr Metab Care.

[20] Roe AJ, Zhang S, Bhadelia RA, Johnson EJ, Lichtenstein AH, Rogers GT (2017). Choline and its metabolites are differently associated with cardiometabolic risk factors, history of cardiovascular disease, and MRI-documented cerebrovascular disease in older adults. Am J Clin Nutr.

[21] Millard HR, Musani SK, Dibaba DT, Talegawkar SA, Taylor HA, Tucker KL (2018). Dietary choline and betaine; associations with subclinical markers of cardiovascular disease risk and incidence of CVD, coronary heart disease and stroke: the Jackson Heart Study. Eur J Nutr.

[22] Martínez-Reyes I, Chandel NS (2020). Mitochondrial TCA cycle metabolites control physiology and disease. Nat Commun.

[23] Bulló M, Papandreou C, García-Gavilán J, Ruiz-Canela M, Li J, Guasch-Ferré M (2021). Tricarboxylic acid cycle related-metabolites and risk of atrial fibrillation and heart failure. Metabolism.

[24] Remchak M-ME, Heiston EM, Ballantyne A, Dotson BL, Stewart NR, Spaeth AM (2022). Insulin sensitivity and metabolic flexibility parallel plasma TCA levels in early chronotype with metabolic syndrome. J Clin Endocrinol Metab.

[25] Dick BP, Yousif A, Raheem O, Hellstrom WJG (2021). Does lowering hemoglobin A1c reduce Penile Prosthesis infection: a systematic review. Sex Med Rev.

[26] Vallejo M, García A, Tuñón J, García-Martínez D, Angulo S, Martin-Ventura JL (2009). Plasma fingerprinting with GC-MS in acute coronary syndrome. Anal Bioanal Chem.

[27] Yao H, Shi P, Zhang L, Fan X, Shao Q, Cheng Y (2010). Untargeted metabolic profiling reveals potential biomarkers in myocardial infarction and its application. Mol Biosyst.

[28] Lin W, Wang M, Chen M, Zheng X, Wu Y, Gao D (2020). Metabolomics and correlation network analyses of core biomarkers in type 2 diabetes. Amino Acids.

[29] Ueland PM, Midttun O, Windelberg A, Svardal A, Skålevik R, Hustad S (2007). Quantitative profiling of folate and one-carbon metabolism in large-scale epidemiological studies by mass spectrometry. Clin Chem Lab Med.

[30] Kumar K, Venkatraman V, Bruheim P (2021). Adaptation of central metabolite pools to variations in growth rate and cultivation conditions in Saccharomyces cerevisiae. Microb Cell Fact.

[31] Liu Z, Tu M-J, Zhang C, Jilek JL, Zhang Q-Y, Yu A-M (2019). A reliable LC-MS/MS method for the quantification of natural amino acids in mouse plasma: Method validation and application to a study on amino acid dynamics during hepatocellular carcinoma progression. J Chromatogr B Analyt Technol Biomed Life Sci.

[32] Guiraud SP, Montoliu I, Da Silva L, Dayon L, Galindo AN, Corthésy J (2017). High-throughput and simultaneous quantitative analysis of homocysteine-methionine cycle metabolites and co-factors in blood plasma and cerebrospinal fluid by isotope dilution LC-MS/MS. Anal Bioanal Chem.

[33] Fu J, Wang T, Liu J, Wang X, Zhang Q, Li M (2019). Using clinical indices to Distinguish MODY2 (GCK mutation) and MODY3 (HNF1A mutation) from type 1 diabetes in a Young Chinese Population. Diabetes Ther.

[34] Bernini P, Bertini I, Luchinat C, Tenori L, Tognaccini A (2011). The cardiovascular risk of healthy individuals studied by NMR metabonomics of plasma samples. J Proteome Res.

[35] Tian Q, Zhao J, Yang Q, Wang B, Deavila JM, Zhu M-J (2020). Dietary alpha-ketoglutarate promotes beige adipogenesis and prevents obesity in middle-aged mice. Aging Cell.

[36] Yuan Y, Zhu C, Wang Y, Sun J, Feng J, Ma Z (2022). α-Ketoglutaric acid ameliorates hyperglycemia in diabetes by inhibiting hepatic gluconeogenesis via serpina1e signaling. Sci Adv.

[37] Hochachka PW, Dressendorfer RH (1976). Succinate accumulation in man during exercise. Eur J Appl Physiol Occup Physiol.

[38] Sadagopan N, Li W, Roberds SL, Major T, Preston GM, Yu Y (2007). Circulating succinate is elevated in rodent models of hypertension and metabolic disease. Am J Hypertens.

[39] He W, Miao FJP, Lin DCH, Schwandner RT, Wang Z, Gao J (2004). Citric acid cycle intermediates as ligands for orphan G-protein-coupled receptors. Nature.

[40] Keiran N, Ceperuelo-Mallafré V, Calvo E, Hernández-Alvarez MI, Ejarque M, Núñez-Roa C (2019). SUCNR1 controls an anti-inflammatory program in macrophages to regulate the metabolic response to obesity. Nat Immunol.

[41] Lumeng CN, DelProposto JB, Westcott DJ, Saltiel AR (2008). Phenotypic switching of adipose tissue macrophages with obesity is generated by spatiotemporal differences in macrophage subtypes. Diabetes.

[42] Menezo Y, Elder K, Clement A, Clement P, Folic, Acid. Folinic Acid, 5 Methyl TetraHydroFolate supplementation for mutations that affect Epigenesis through the Folate and One-Carbon cycles. Biomolecules. 2022;12(2). 10.3390/biom12020197 PMC8961567 35204698

[43] Wang S, Li M, Lin H, Wang G, Xu Y, Zhao X (2022). Amino acids, microbiota-related metabolites, and the risk of incident diabetes among normoglycemic chinese adults: findings from the 4 C study. Cell Rep Med.

[44] Hosseinkhani S, Arjmand B, Dilmaghani-Marand A, Mohammadi Fateh S, Dehghanbanadaki H, Najjar N (2022). Targeted metabolomics analysis of amino acids and acylcarnitines as risk markers for diabetes by LC-MS/MS technique. Sci Rep.

[45] Homocysteine (2002). Risk of ischemic heart disease and stroke: a meta-analysis. JAMA.

[46] Atabek ME, Pirgon O, Karagozoglu E (2006). Plasma homocysteine levels in children and adolescents with type 1 diabetes. Indian Pediatr.

[47] Cronin CC, McPartlin JM, Barry DG, Ferriss JB, Scott JM, Weir DG (1998). Plasma homocysteine concentrations in patients with type 1 diabetes. Diabetes Care.

[48] Arroyo-Jousse V, García-Díaz DF, Pérez-Bravo F (2015). [Global DNA methylation and homocysteine levels are lower in type 1 diabetes patients]. Rev Med Chil.

[49] Mentch SJ, Mehrmohamadi M, Huang L, Liu X, Gupta D, Mattocks D (2015). Histone methylation Dynamics and Gene Regulation occur through the sensing of One-Carbon Metabolism. Cell Metab.

[50] Holm LJ, Haupt-Jorgensen M, Larsen J, Giacobini JD, Bilgin M, Buschard K (2018). L-serine supplementation lowers diabetes incidence and improves blood glucose homeostasis in NOD mice. PLoS ONE.

[51] Guasch-Ferré M, Hruby A, Toledo E, Clish CB, Martínez-González MA, Salas-Salvadó J (2016). Metabolomics in Prediabetes and Diabetes: a systematic review and Meta-analysis. Diabetes Care.

[52] Gaggini M, Carli F, Rosso C, Buzzigoli E, Marietti M, Della Latta V (2018). Altered amino acid concentrations in NAFLD: impact of obesity and insulin resistance. Hepatology.

[53] Dollet L, Kuefner M, Caria E, Rizo-Roca D, Pendergrast L, Abdelmoez AM (2022). Glutamine regulates skeletal muscle immunometabolism in type 2 diabetes. Diabetes.

[54] Leiva-Gea I, Sánchez-Alcoholado L, Martín-Tejedor B, Castellano-Castillo D, Moreno-Indias I, Urda-Cardona A (2018). Gut microbiota differs in composition and functionality between children with type 1 diabetes and MODY2 and healthy control subjects: a case-control study. Diabetes Care.

