# Supplementary material for: Quantitative profiling and diagnostic potential of one-carbon and central metabolism pools in MODY2 and T1DM
Source: Diabetol Metab Syndr. 2023 Oct 24;15:206. doi: 10.1186/s13098-023-01175-x (PMC10594937; doi:10.1186/s13098-023-01175-x)
Supplement: Supplementary file 1 — Supplementary Material 1 [file 13098_2023_1175_MOESM1_ESM.docx]

**Supplementary materials**

**Table S1. List of mutations of MODY2 (GCK) patients.**

| **Subject** | **Location** | **c.DNA** | **Type** | **Reported or not** |
| --- | --- | --- | --- | --- |
| G1 | Exon 7 | c.683C>T | missense | Yes |
| G2 | Exon 7 | c.686C>T | missense | Yes |
| G3 | Exon 7 | c.686C>T | missense | Yes |
| G4 | Exon 6 | c.667G>A | missense | Yes |
| G5 | Exon 2 | c.130G>A | missense | Yes |
| G6 | Exon 2 | c.130G>A | missense | Yes |
| G7 | Exon 5 | c.556C>T | missense | Yes |
| G8 | Exon 5 | c.556C>T | missense | Yes |
| G9 | Exon 7 | c.755G>A | missense | Yes |
| G10 | Exon 7 | c.755G>A | missense | Yes |
| G11 | Exon 5 | c.571C>T | missense | Yes |
| G12 | Exon 4 | c.502A>G | missense | Yes |
| G13 | Exon 9 | c.1223G>T | missense | Yes |
| G14 | Exon 6 | c.584T>C | missense | Yes |
| G15 | Exon 6 | c.584T>C | missense | No |
| G16 | Exon 6 | c.584T>C | missense | No |
| G17 | Exon 5 | c.556C>T | missense | Yes |
| G18 | Exon 5 | c.556C>T | missense | Yes |
| G19 | Exon 5 | c.556C>T | missense | Yes |
| G20 | Exon 5 | c.556C>T | missense | Yes |
| G21 | Exon 5 | c.556C>T | missense | Yes |
| G22 | Exon 5 | c.556C>T | missense | Yes |
| G23 | Exon 8 | c.898G>A | missense | Yes |
| G24 | Exon 5 | c.553C>T | missense | No |
| G25 | Exon 7 | c.803_804insA | insertion | No |
| G26 | Exon 3 | c.263T>G | missense | No |
| G27 | Exon 3 | c.263T>G | missense | No |
| G28 | Exon 4 | c.370G>C | missense | Yes |
| G29 | Exon 4 | c.370G>C | missense | Yes |
| G30 | Exon 5 | c.532_532delG | deletion | Yes |
| G31 | Exon 4 | c.532_532delG | deletion | Yes |
| G32 | Exon 4 | c.452_454delCCT | deletion | Yes |
| G33 | Exon 1 | c.121G>A | missense | Yes |

| **Pathogenicity assessment of first-reported GCK mutation using software** | | | |
| --- | --- | --- | --- |
| **Mutation** | **Mutation taster** | **SIFT** | **Polyphen-2** |
| c.263T>G | Disease causing | Deleterious | probably damaging(1.000) |
| c.553C>T | Disease causing | Deleterious | probably damaging(1.000) |
| c.584T>C | Disease causing | Deleterious | probably damaging (1.000) |
| c.803_804insA | Disease causing | Neutral | possibly damaging (0.917) |

In this study, patients with MODY2 carried 18 gene mutations, including 15 missense mutations, 2 deletion mutations and 1 insertion mutation. Consistent with previous studies, most mutations are in the 2-10 Exon of GCK gene. There are four mutations reported for the first time, which are in the Exon 3 (c.263T>G), 5 (c.553C>T), 6 (c.584T>C) and 7 (c.803_804insA) of GCK.

The above four mutations were predicted to be pathogenic mutations by three kinds of software, of which the first three missense mutation are reported harmful mutations by Mutation taster, SIFT and Polyphen-2 function prediction software; and the insertion mutation (c.803_ 804insA) is predicted to be a harmful mutation through Mutation taster and Polyphen-2, combined with the phenomenon of family co segregation, according to ACMG standards, it conforms to pathogenic mutations.

**Table S2. Optimized liquid chromatography and MS conditions and calibration curves information of individual metabolites.**

| No. | analyte | DP(V) | CE(eV) | transition | regression equation | R2 |
| --- | --- | --- | --- | --- | --- | --- |
| 1 | Glycine | 30 | 19 | 76.0-30.0 | y=0.206x+-0.0000401 | 0.996 |
| 2 | Trimethylamine | 70 | 15 | 76.1-58.0 | y=138.293x+0.0267 | 0.992 |
| 3 | Serine | 74 | 25 | 106.0-60.0 | y=1.61x+0.00538 | 0.991 |
| 4 | Betaine | 56 | 29 | 118.1-58.0 | y=340x+0.365 | 0.992 |
| 5 | Threonine | 52 | 19 | 120.1-74.0 | y=7.24x+0.00183 | 0.992 |
| 6 | Cysteine | 66 | 25 | 122.0-59.0 | y=0.132x+0.00439 | 0.99 |
| 7 | Glutamic acid | 80 | 35 | 148.1-84.0 | y=0.130x+0.0408 | 0.995 |
| 8 | Methionine | 72 | 19 | 150.1-104.0 | y=2.99x+0.00448 | 0.992 |
| 9 | Cystathionine | 70 | 41 | 223.1-88.0 | y=14.5x+0.00188 | 0.993 |
| 10 | SAH | 64 | 35 | 385.1-136.0 | y=3.01x+-0.00236 | 0.993 |
| 11 | SAM | 80 | 21 | 399.0-136.0 | y=-0.00149x+0.000382 | 0.995 |
| 12 | Hcy | 80 | 35 | 136.1-47.0 | y=-0.000187x+0.0000434 | 0.991 |
| 13 | Fumaric acid | -80 | -15 | 115.0-71.0 | y=0.116x+0.000306 | 0.992 |
| 14 | Succinic acid | -70 | -15 | 117.0-73.0 | y=0.073x+0.000228 | 0.99 |
| 15 | α-KG | -80 | -19 | 145.0-99.0 | y=-0.0031x+0.000819 | 0.991 |
| 16 | Glutamine | -80 | -19 | 145.1-127.0 | y=0.00243x+0.0000434 | 0.99 |
| 17 | pyruvate | -70 | -15 | 87.0-43.0 | y=0.491x+0.0552 | 0.991 |
| 18 | citrate | -80 | -20 | 191.0-129.0 | y=1.42x+0.00231 | 0.99 |
| 19 | lactic | -70 | -25 | 89.0-41.0 | y=109x+0.0275 | 0.991 |
| 20 | malate | -70 | -15 | 133.0-115.0 | y=2.38x+0.0135 | 0.994 |
| 21 | oxaloacetate | -70 | -15 | 131.0-87.0 | y=0.0996x+0.000484 | 0.993 |

**Figure S1. PLS-DA score plot for metabolites in three groups.**


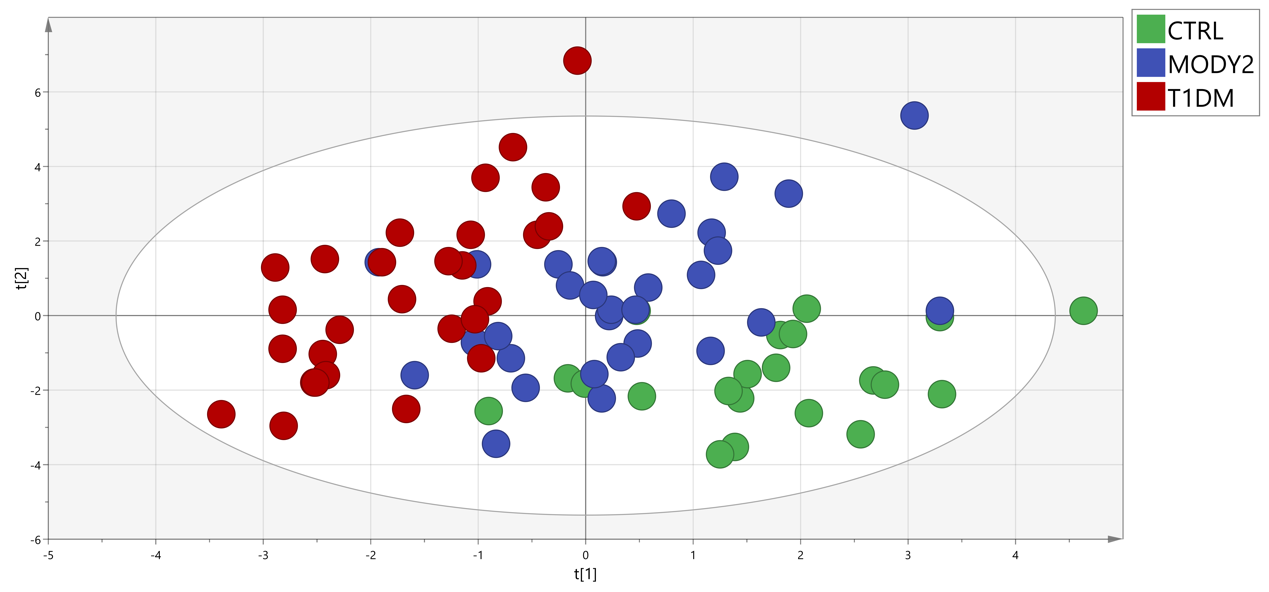


R2X = 0.448, R2Y = 0.382, Q2 = 0.294

**Figure S2. Metabolites in 4 groups after stratified analysis.**


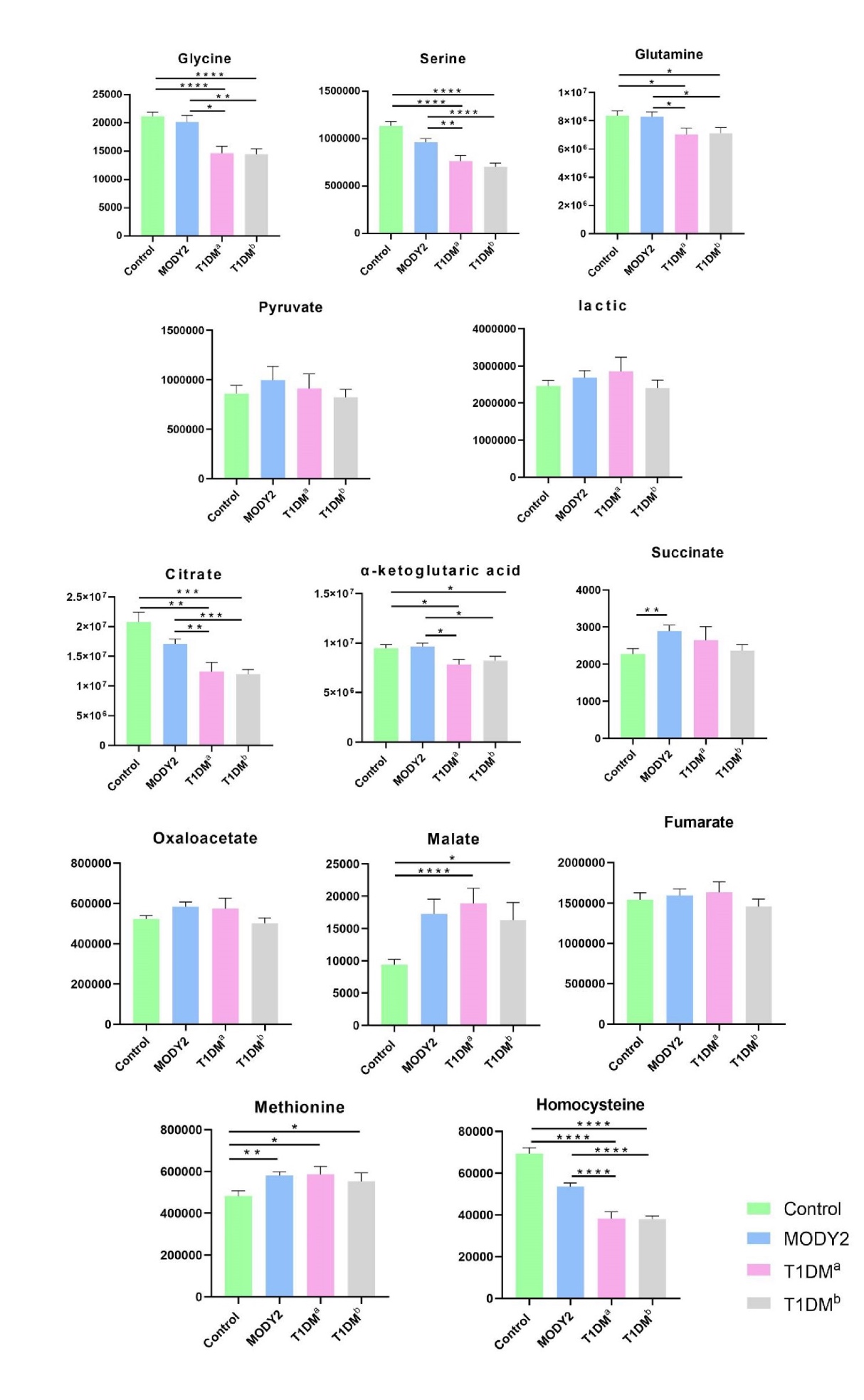


T1DM^a^: well-controlled T1DM; T1DM^b^: poor blood glucose control.

**The blood glucose parameters in 4 groups after stratified analysis.**

| Parameters | Control | MODY2 | T1DM^a^ | T1DM^b^ | *P* |
| --- | --- | --- | --- | --- | --- |
| Fasting glucose (mmol/L) | 4.8 ± 0.37 | 6.8 ± 0.57 | 6.3 ± 0.97 | 10.9 ± 3.9***** | **<0.0001** |
| HbA1c (%) | 4.7 ± 0.4 | 6.3 ± 0.37 | 6.9 ± 1.39 | 10.1 ± 2.03***** | **<0.0001** |

The data were expressed as mean ± standard deviation after one-way ANOVA;

P < 0.05 indicates a significant difference, which is indicated in bold; differences from MODY2 are indicated by *

There is no difference in fasting glucose and HbA1c between MODY2 and T1DM^a^ groups.
